# Supplementary figures and images for: Quantifying immune-based counterselection of somatic mutations
Source: PLoS Genet. 2019 Jul 25;15(7):e1008227. doi: 10.1371/journal.pgen.1008227 (PMC6657826; doi:10.1371/journal.pgen.1008227)

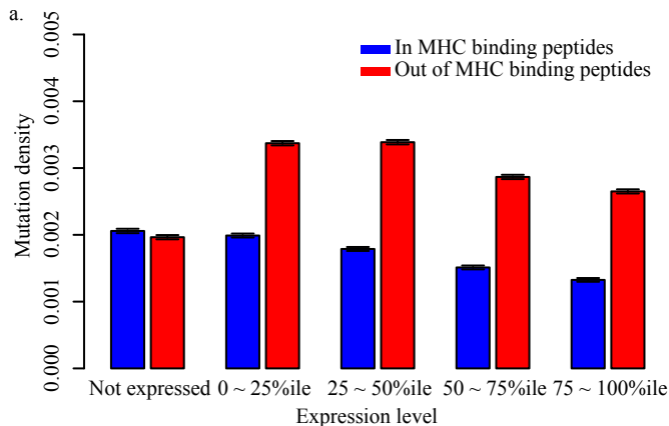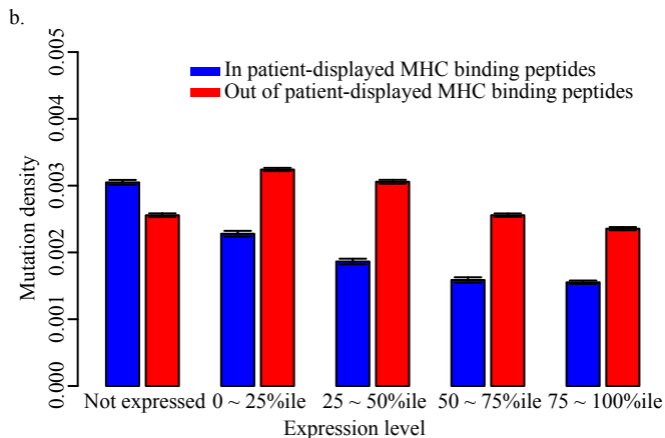

Supplement: S1 Fig — a. MHC-display-dependent mutation densities for genes with different expression levels using TCGA dataset. Blue bars indicate mutation density within the predicted MHC-binding peptides. Red bars are the mutation density out of the predicted MHC-binding peptides. Mutations were separated into five categories based on the expression levels of their genes. b. MHC-display-dependent mutation densities for genes with different expression levels, considering each TCGA patient’s HLA type. Blue bars are the mutation density within the predicted patient-displayed MHC binding peptides. Red bars are the mutation density out of the patient-displayed predicted MHC binding peptides. Mutations were separated into five categories based on the expression levels of their genes. (PDF) [file pgen.1008227.s001.pdf]

a.

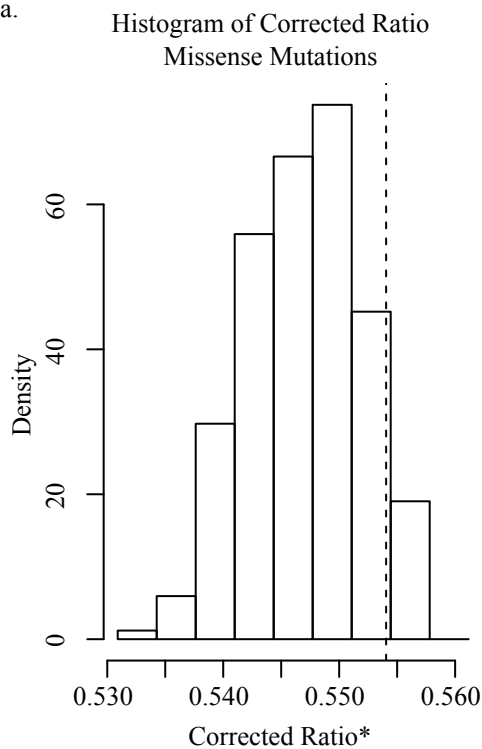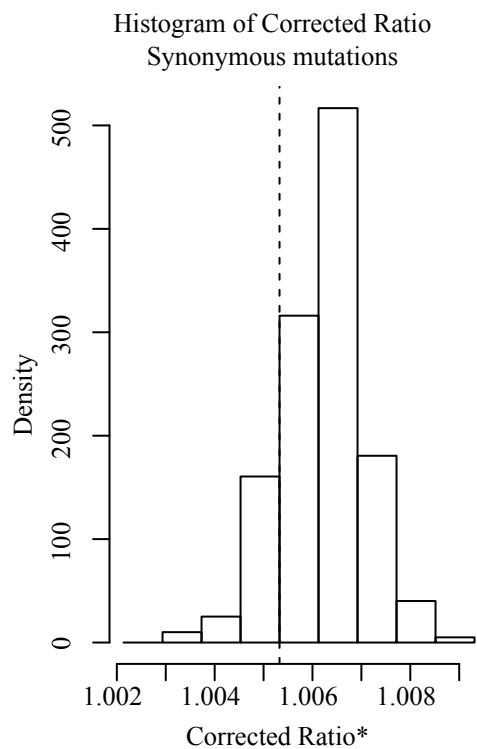

b.

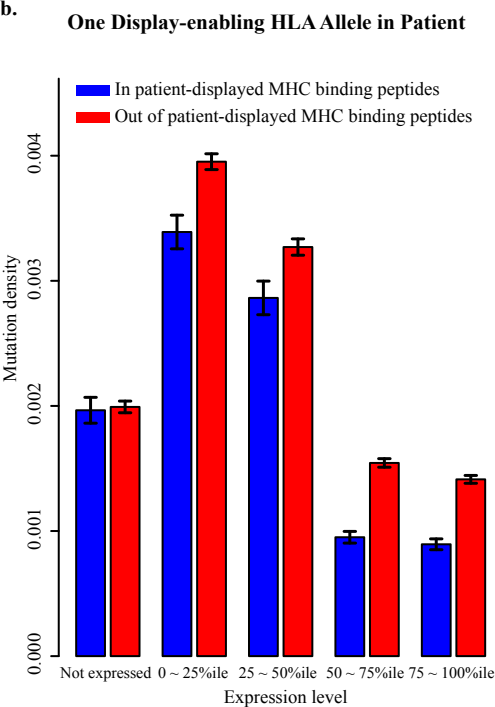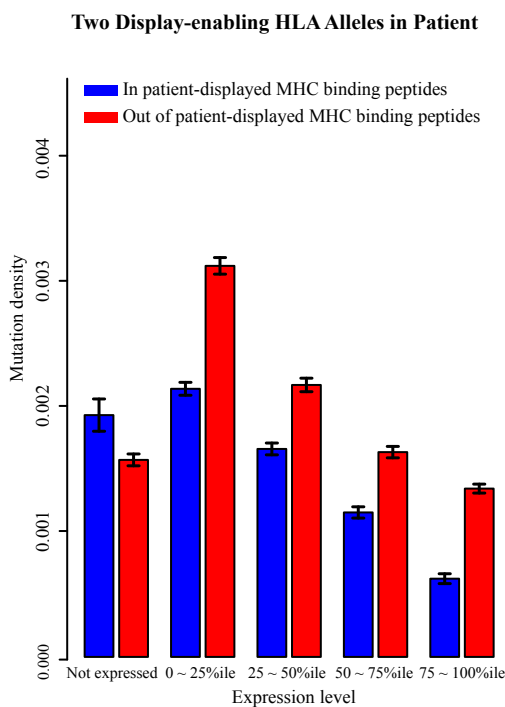

Supplement: S2 Fig — a. Exploring uncertainty in corrected mutation density ratio for TCGA mutations in patient-displayed MHC binding peptides. Bootstrap resampling was used for both missense variants (left panel) and synonymous variants (right panel) Observed values are indicated with a vertical dashed line. b. MHC-display-dependent mutation densities for genes with different expression levels, considering the number of displaying HLA alleles. Average mutation density in peptides predicted to be displayed by one or two of the 12 common HLA-A or HLA-B allele types. A. Mutation density in peptides predicted to be displayed in patients by only one HLA allele. B. Mutation density in peptides predicted to be displayed in patients with two displaying HLA alleles. (PDF) [file pgen.1008227.s002.pdf]

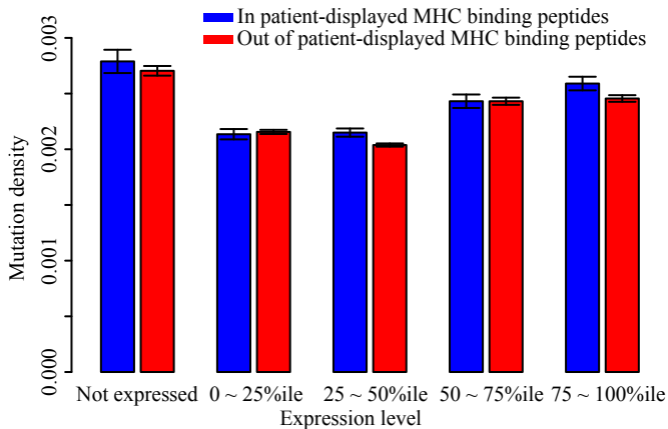

Supplement: S3 Fig — Blue bars are the synonymous mutation density within the predicted patient-displayed MHC binding peptides. Red bars are the synonymous mutation density out of the patient-displayed predicted MHC binding peptides. Synonymous mutations were separated into five categories based on the expression levels of their genes. (PDF) [file pgen.1008227.s003.pdf]

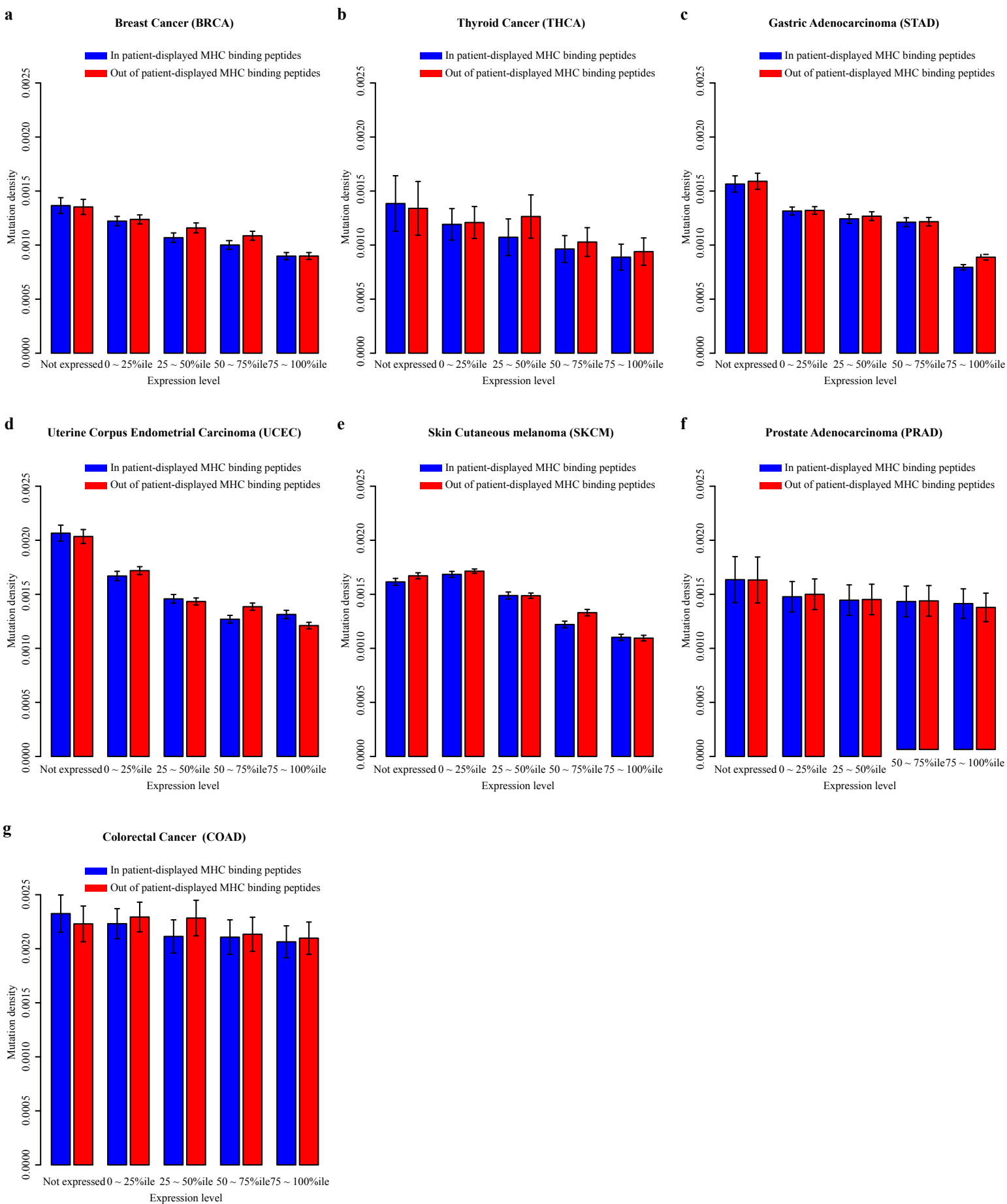

Supplement: S4 Fig — Blue bars are the mutation density within the predicted patient-displayed MHC binding peptides. Red bars are the mutation density out of the patient-displayed predicted MHC binding peptides. Mutations were separated into five categories based on the expression levels of their genes. (PDF) [file pgen.1008227.s004.pdf]

**a****One Display-enabling HLA Allele in Patient**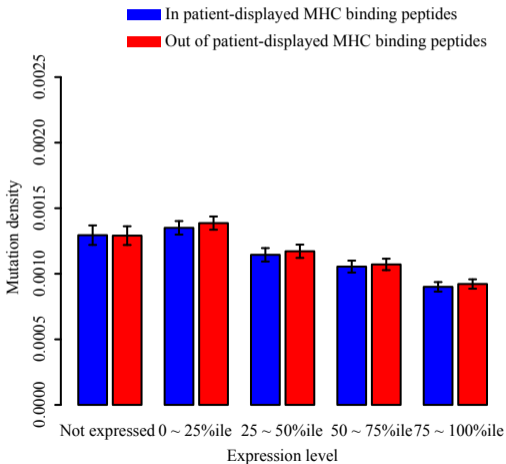**b****Two Display-enabling HLA Alleles in Patient**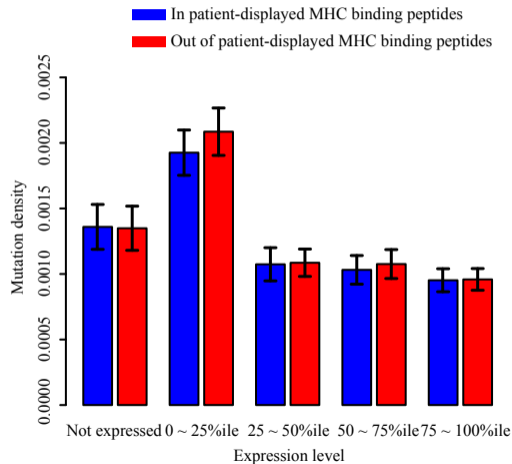

Supplement: S5 Fig — Average mutation density in peptides predicted to be displayed by one or two of the 12 common HLA-A or HLA-B allele types. A. Mutation density in peptides predicted to be displayed in patients by only one HLA allele. B. Mutation density in peptides predicted to be displayed in patients with two displaying HLA alleles. (PDF) [file pgen.1008227.s005.pdf]
