# Supplementary material for: Quantifying immune-based counterselection of somatic mutations
Source: PLoS Genet. 2019 Jul 25;15(7):e1008227. doi: 10.1371/journal.pgen.1008227 (PMC6657826; doi:10.1371/journal.pgen.1008227)
Supplement: S1 Table — (PDF) [file pgen.1008227.s006.pdf]

S1 Table. List of 37 Different PCAWG Cancer Types with Number of Samples and Mutated Genes of Each Cancer Type

Cancer	Number of Samples	Number of Mutated Genes
BLCA	23	4004
BOCA	61	1327
BRCA	207	10304
BTCA	11	719
CESC	20	956
CLLE	100	1293
CMDI	48	433
COAD	46	37666
DLBC	7	744
EOPC	41	771
ESAD	39	4787
GACA	27	2000
GBM	41	2604
HNSC	42	4899
KICH	49	1112
KIRC	40	2162
KIRP	34	1613
LAML	37	353
LGG	19	336
LICA	6	505
LIHC	52	3446
LIRI	39	2465
LUAD	40	7781
LUSC	48	10980
MALY	97	6479
ORCA	12	879
OV	114	6946
PACA	140	8675
PAEN	88	1846
PBCA	215	2057
PRAD	40	708
READ	16	14882
SARC	33	1179
SKCM	36	21278
STAD	39	10152

THCA	48	527
UCEC	50	19460
