# Supplementary material for: Quantifying immune-based counterselection of somatic mutations
Source: PLoS Genet. 2019 Jul 25;15(7):e1008227. doi: 10.1371/journal.pgen.1008227 (PMC6657826; doi:10.1371/journal.pgen.1008227)
Supplement: S2 Table — (PDF) [file pgen.1008227.s007.pdf]

S2 Table. List of 31 Different TCGA Cancer Types with Number of Samples and Mutated Genes of Each Cancer Type

Cancer	Number of Samples	Number of Mutated Genes
ACC	90	10805
BLCA	124	22991
BRCA	973	51806
CESC	149	21538
CHOL	35	3758
COAD	60	7305
COADREAD	60	7305
DLBC	14	1186
ESCA	181	32197
GBM	205	9340
HNSC	178	19679
KIRP	158	8861
LAML	109	815
LIHC	170	15863
LUAD	163	30987
LUSC	110	24512
PAAD	98	14334
PCPG	184	2128
PRAD	329	7723
SARC	238	10308
SKCM	341	161952
STAD	275	75745
TGCT	154	7598
THCA	386	4339
THYM	112	1553
UCEC	240	113697
UCS	7	7
UVM	8	8
UCS	57	6512
UVM	80	1319
